# Chimeric Mouse Model for the Infection of Hepatitis B and C Viruses 

**DOI:** 10.1371/journal.pone.0077298

**Published:** 2013-10-14

**Authors:** Abeba Tesfaye, Judith Stift, Dragan Maric, Qingwen Cui, Hans-Peter Dienes, Stephen M. Feinstone

**Affiliations:** 1 Division of Viral Products, Center for Biologics Evaluation and Research, FDA, Bethesda, Maryland, United States of America; 2 Institute of Pathology, Medical University of Vienna, Wien, Austria; 3 Division of Intramural Research, Flow Cytometry Core Facility, National Institute of Neurological Disorders and Stroke, National Institutes of Health, Bethesda, Maryland, United States of America; Saint Louis University, United States of America

## Abstract

While the chimpanzee remains the only animal that closely models human hepatitis C virus (HCV) infection, transgenic and immunodeficient mice in which human liver can be engrafted serve as a partial solution to the need for a small animal model for HCV infection. The established system that was based on mice carrying a transgene for *urokinase-type plasminogen activator (uPA*) gene under the control of the human albumin promoter has proved to be useful for studies of virus infectivity and for testing antiviral drug agents. However, the current *Alb-uPA* transgenic model with a humanized liver has practical limitations due to the inability to maintain non-engrafted mice as dizygotes for the transgene, poor engraftment of hemizygotes, high neonatal and experimental death rates of dizygous mice and a very short time window for hepatocyte engraftment. To improve the model, we crossed transgenic mice carrying the *uPA* gene driven by the major urinary protein promoter onto a *SCID/Beige* background (*MUP-uPA SCID/Bg*). These transgenic mice are healthy relative to *Alb-uPA* mice and provide a long window from about age 4 to 12 months for engraftment with human hepatocytes and infection with hepatitis C or hepatitis B (HBV) viruses. We have demonstrated engraftment of human hepatocytes by immunohistochemistry staining for human albumin (30-80% engraftment) and observed a correlation between the number of human hepatocytes inoculated and the level of the concentration of human albumin in the serum. We have shown that these mice support the replication of both HBV and all six major HCV genotypes. Using HBV and HCV inocula that had been previously tittered in chimpanzees, we showed that the mice had approximately the same sensitivity for infection as chimpanzees. These mice should be useful for isolating non-cell culture adapted viruses as well as testing of antiviral drugs, antibody neutralization studies and examination of phenotypic changes in viral mutants.

## Introduction

Hepatitis C is a major public health problem that affects an estimated 180 million people worldwide. In the US alone, there are nearly 3 million HCV infected patients [1]. Chronic HCV infected patients are at risk of developing chronic liver disease, cirrhosis and eventually liver cancer [2-4]. The virus has a single strand, plus sense RNA genome with an envelope derived from host cellular membranes. There are six major genotypes of the hepatitis C virus with additional subtypes [5] but it is not known if these genotypes also relate to serotype diversity. The virus has a limited host range of humans and chimpanzees and the chimpanzee remains the only complete animal model for HCV infection and disease that can be used in studies of pathogenesis of hepatitis C virus and immune response to infection or for preclinical evaluation of developmental vaccines [6,7]. While there is no vaccine to prevent hepatitis C virus infections [8], antiviral treatment with alpha interferon and ribavirin is effective in actually curing the infection in up to fifty percent of patients with chronic HCV. The addition of newer, direct acting antiviral agents can improve the outcome of treatment to over eighty percent [9-11].

Over the past few years, several transgenic mouse models have been developed that support the replication of HBV and HCV. The successful infection of chimeric mice in which the diseased mouse livers were repopulated by human hepatocytes was reported beginning in 2001 [12-14]. These immunodeficient mice (*SCID/Bg*) carry an array of tandem copies of the transgene for urokinase plasminogen activator (uPA) under the control of the albumin promoter (*Alb-uPA*). The Alb-uPA mice must be maintained as hemizygotes for the transgene because dizygous animals are severely impaired with short survival windows and adult survivors breed poorly unless engrafted with normal liver cells [15]. However, hemizygous mice did not achieve a high level of liver injury and therefore transplanted human hepatocytes did not adequately engraft the mice at levels that would support robust replication of HCV. Several other systems similar to the *Alb-uPA* mice have recently been reviewed in 7. Recently, excellent levels of liver repopulation by human hepatocytes was demonstrated in severely immuno-deficient (*Rag2* ^*-*^ */* ^*-*^ *Il2rg* ^*-*^ */* ^*-*^) fumarylacetoacetate hydrolase (Fah)-deficient mouse [16]. These, triply mutant, mice are maintained by adding a drug, 2-(2-nitro-4-trifluromethylbenzoyl)-1, 3-cyclohexanedione (NTBC) into their diet to prevent accumulation of toxic metabolites in the liver that can cause hepatic destruction [17]. Withdrawal of the drug results in hepatic injury and the mice can be efficiently engrafted with human hepatocytes [18,19].

Here, we describe, immuno-deficient mice that have the *uPA* gene under the control of the major urinary protein (MUP) promoter [20,21]. The *MUP-uPA/SCID/Bg* transgenic mice can be efficiently and consistently repopulated with human hepatocytes and support the replication of hepatitis B virus and hepatitis C including all six major genotypes as well as tissue culture-derived virus. We have optimized a protocol for reconstituting the mouse liver with fresh human hepatocytes and determined the time course for infection with the virus. Furthermore, we have determined the infectivity titers of both patient-derived HCV and HBV isolates in these chimeric mice and compared those titers to the historical titers of the same inocula in chimpanzees.

## Materials and Methods

### Generation of MUP-*uPA/SCID/Beige* transgenic mice

SCID/Bg transgenic mice expressing the secreted form of human urokinase plasminogen activator (uPA) were previously described [20,21]. The *MUP-uPA* transgene construct contains the *MUP enhancer/promoter*, the entire mouse uPA genomic coding sequence [20]. *MUP-uPA* mice were crossed with *SCID/Beige* background Balb/c mice [21]. Transgenic mice offspring were identified by PCR, using forward primers specific for *uPA*, 5′-GCGATTCTGGAGGACCGCTTATC-3′, 5′-TTAGGACAAGGCTGGTGGGCACTG-3′. Twenty-five μL of reaction mixture containing 200 ng of genomic DNA extracted from a tail snip was subjected to the following conditions: 1) 92°C for 2 minutes; 2) 35 cycles of: 45 seconds at 92°C, 1 minute at 60°C, and 1 minute at 72°C; and 3minutes at 72°C for 5 minutes. An amplified product from the *uPA* transgene showed a 300 bp band on a 2% agarose gel.

### Transplantation of human hepatocytes in *MUP-uPA/SCID/Bg* mice

All human hepatocyte transplantation procedures performed on the animals were approved by the Center for Biologics Evaluation and Research/FDA Institutional Animal Care and Use Committee (CBER/IACUC). Primary human hepatocytes (CellzDirect, USA) were shipped by overnight express. Fresh hepatocytes were transplanted immediately upon arrival within 12-16 hour after isolation. Viable cell counts were determined by trypan blue exclusion. Initially, mice were injected with less than 1 x 10^6^ cells, but as we optimized the system, the number of cells was increased to 4-6 x 10^6^ hepatocytes and suspended in 200 uL of normal saline solution (Vedco, St. Joseph, MO). An approximate 1 cm skin incision was made in the upper left quadrant of the mouse abdomen leaving the peritoneum intact. By moving the incision around over the peritoneal membrane, the spleen could be visualized. Using a 27-gauge needle, 200 uL of the cell suspension were injected directly into the spleen. The incision was then closed with a drop of Vetbond tissue adhesive (3M Animal Care Products, St. Paul, MN). Similarly, control mice were injected intra-splenically with 200 uL of normal saline solution. 

### Human albumin measurement

Small amounts of blood were collected weekly from the tail vein and the serum was collected after centrifugation. After 1,000 and 10,000 dilutions of the serum with Tris-buffered saline, human albumin concentrations were measured with the ELISA Quantitation Kit (Bethyl Laboratories) according to the manufacturer’s protocol.

### Histology

Mice with humanized liver were sacrificed and the livers were removed and fixed with 4% (v/v) phosphate-buffered formalin overnight, processed and embedded in paraffin blocks...Slides containing representative sections of the liver were prepared and stained with hematoxylin and eosin (H&E) or prepared for immune-staining. Liver tissues from non-engrafted *MUP-uPA/SCID/Bg* mice were prepared in the same manner to be used as comparative controls..

### HCV and HBV infection of MUP-*uPA/SCID/Bg* mice

*MUP-uPA/SCID/Bg* mice that had been engrafted with human hepatocytes were inoculated intravenously (i.v.) through the tail vein with 100 uL of diluted plasma from a HCV-infected chimpanzee containing approximately 30 chimpanzee infectious doses (CID_50_) per mL of genotype 1-a HCV strain H or 100 chimpanzee infectious dose per mL of clinical isolates of HCV genotype 1a, 2a, 3a, 4a, 5a and 6a that had been passaged in chimpanzees [22]. Several ten-fold dilutions (10^-2^ to 10^-8^) of chimpanzee serum containing 10^7.5^ infectious doses per mL of HBV (subtype ayw) were prepared, and engrafted *MUP-uPA/SCID/Bg* mice were inoculated with 100 uL of these HBV dilutions intravenously [23]. Both the HCV genotype inocula and the HBV titered inoculum were gifts from Dr. Robert Purcell (Laboratory of Infectious Diseases, National Institute of Allergy and Infectious Diseases, National Institute of Health). In addition, 100 uL of HCV cell culture (HCVcc) supernatant containing 10^4^ Fluorescent focus units (ffu) from J6/JFH1 [24] infected Huh 7.5 cells were used to inoculate mice as previously described. Control mice were inoculated with Hank’s buffer containing 0.5% human serum. All viral infections were carried out using an approved protocol specific for this study by the CBER/IACUC.

### Immunohistochemistry

Human cells were specifically stained using HRP-conjugated goat anti-human albumin (Bethyl Laboratories) as primary antibody at dilution of 1:50, blocked with 5% goat serum followed by 3, 3'-diaminobenzidine (DAB) chromagen staining and counterstained with hematoxylin according to the manufacturer’s specifications. Briefly, at least 10 areas were selected and all the nuclei as well as the human albumin positive cells were counted by eye. The percentage of human albumin positive cells in each sample was determined by dividing the mean value of human albumin positive cells into the mean value of all cells in the sample slide.

For detection of HCV and HBV, we used primary goat anti-HCV (NS5) (1:200; Abcam Cat. # 20773) that targets NS5A and NS5B genotype 1a or rabbit anti-HBV core antigen (1:200; Cell Marque) followed by a red fluorescent labeled secondary donkey anti goat IgG or donkey anti-rabbit IgG at dilution of 1:400 (Alexa Fluor 647) Molecular Probes, Inc.). To localize virus antigens in the engrafted human hepatocytes, we used dual staining with rabbit anti-human albumin as primary antibody at dilution 1:500 (Bethyl Laboratories) and fluorescent-labeled donkey anti-rabbit IgG (Alexa Fluor 488) and the appropriate anti-HCV or anti-HBV with labeled secondary antibodies (Alexa Fluor 647) as described above. Samples were incubated overnight at 4°C with the primary antibodies and after rinsing, incubated for 1 hour at room temperature with the secondary antibodies. Positive cells in the liver sections were observed using an inverted microscope Axiovert 200.

### HCV RNA by real-time qRT-PCR and nested PCR (nPCR)

Sera from the *MUP-uPA/*SCID/Bg mice were collected and RNA prepared for measuring HCV RNA. Similarly, liver tissues were snap-frozen in liquid nitrogen and stored at -80°C for RNA extraction. HCV RNA was isolated using the RNeasy kit (Qiagen). HCV genome copy number was quantified by one-step real-time qRT-PCR reaction using Taqman EZ rTth polymerase kit (Applied Biosystems) in a 25 uL reaction volume with 5 uL of total RNA, 300 uM each of dNTPs, 300 nM of primers, 100 nM of probe, 2.5 mM of Mn(OAc), and 5U of recombinant rTth DNA polymerase. Primer-probe set for qRT-PCR and all primers for nested PCR reactions were previously described [25,26]. For qRT-PCR, reverse transcription and subsequent amplification steps were performed at 50°C for 2 min, followed by 30 min at 60°C and 2 min at 95°C for one cycle, and three cycles at 95°C for 20 s and at 60°C for 30 s followed by 40 cycles of 20 s at 95°C and 1 min at 60°C in the ABI Prism 7900 instrument. The standard curve was generated by using serial dilutions (10^5^ to 10° copies per reaction) of a plasmid containing the HCV genome. Reactions for the standard curve were done in triplicates while experimental samples were analyzed in duplicates. Nested PCR experiments were carried out with a 50 uL reaction containing the first set of primers (300 nM) spanning 47-68 bp (forward) and 263-243 bp (reverse) using 10 uL of total RNA,300 uM each of dNTPs, 2.5 mM Mn(OAc), and 5U of rTth DNA polymerase. In the second reaction, we used a second set of primers (300 nM) that span 86-107 bp (forward) and 227-209 bp (reverse) 300 uM each of dNTPs, 1.25U of Taq gold DNA polymerase and 4 mM MgCl_2_ and 5 uL of the first PCR reaction was used to amplify HCV cDNA. The conditions for RT-PCR were: 1^st^ reaction: 50°C for 60 min followed by 94°C for 2 min and 40 cycles at 94°C for 30 s, 65°C for 30s and 60°C for 60 s and final hold a6 60°C for 7 min. The parameters for the 2^nd^ PCR reaction were 94°C for 2 min, and 40 cycles at 95°C for 30 s 60°C for 3 min and hold at 60°C for 7 min**.**

### HBV genome equivalent copies by real-time qRT-PCR

Mice were bled every 15 days for 4-8 weeks and HBV DNA extracted (Qiagen DNA extraction kit) from mouse serum. Briefly, 10 uL of DNA was subjected to HBV-specific TaqMan PCR in a 50-ul reaction mixture with TaqMan universal PCR master mix and 20X primer/probe mix (Applied Biosystems), 200 nM forward primer HBV469U (5’CCCGTTTGTCCTCTAATTCC-3’), 200 nM reverse primer HBV569L, (5’-GTCCGAAGGTTTGGTACAGC-3’), and 100 nM TaqMan probe HBV495P [5-6-FAMd(CTCAACAACCAGCACGGGACCA)bhq-1-3’)] final concentrations. We used ten-fold dilutions (10^5^ to 10° copies) of plasmid DNA containing HBV insert (generous gift from Dr. Gerardo Kaplan, Division of Emerging Transmitted and Transfusion Diseases, Center for Biologics Evaluation & Research) as standards in parallel with the HBV specific q-PCR reactions.

### Statistical analysis

Statistical significance of differences between groups was tested using with the Kruskal-Wallis tests or one-way ANOVA where appropriate. A probability value of P < 0.05 was considered significant.

## Results

### *MUP-uPA/SCID/Bg* mice

The *MUP-uPA/SCID/Bg* mice used in these experiments were bred and maintained as dizygotes. These animals remained reasonably healthy to, at least, one year of age. Mortality among newborn pups was very low, resembling that observed in *SCID/Bg* mice. Most human hepatocytes engraftments were performed in the mice greater than 4 months of age. Surgical deaths were very infrequent. In multiple experiments, we have engrafted a total of 251 mice and had 23 surgical deaths (9%). The majority of mice (86%) had no visible health issues for several months of observation (Table 1).

**Table 1:** Total number of *MUP-uPA/SCID/Bg* mice engrafted and infected with either HCV or HBV and survival rate.

	HCV-infected	HBV-infected	Control^a^	Total No of mice
Number of mice dead^b^ within a month	20	4	9	33
Number of mice euthanized after 1 month	105	44	46	195
Total number of mice infected	125	48	55	228

a= Mice engrafted but not inoculated with virus.

b= Dead due to poor health.

### Liver damage in response to *MUP-uPA* expression

**Figure 1:**
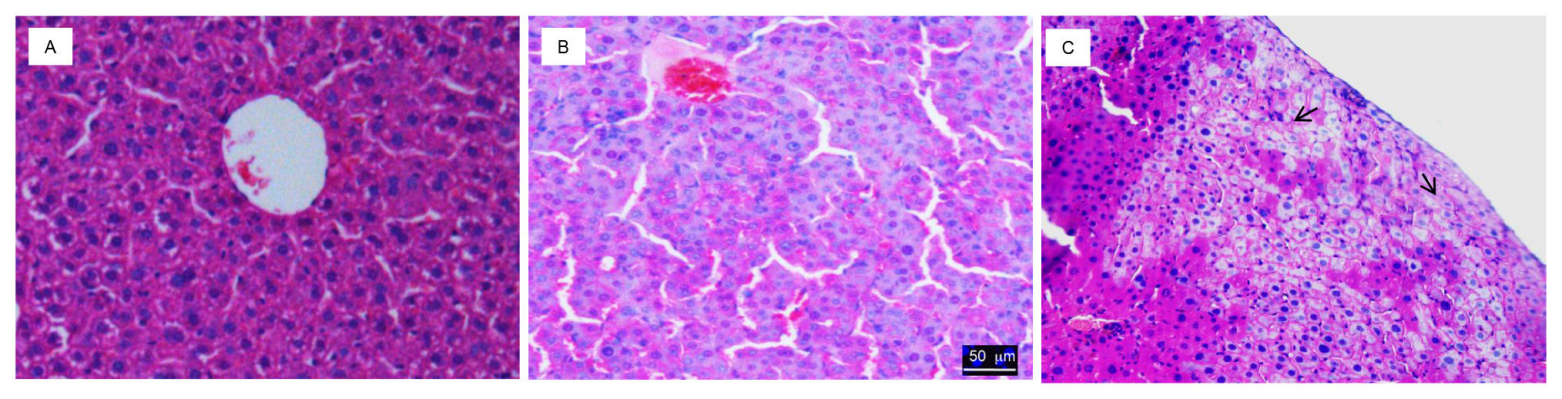
H & E staining of a non-engrafted MUP-uPA-SCID/Bg mouse liver. **A**. Liver section from a 2-m old transgenic mouse with no necrosis or inflammation. **B**. Liver section from a 4-m old transgenic mouse. **C**. Liver section from a 6-m old transgenic mouse shows extensive areas of necrosis (Arrow).

To examine the extent in which expression of the *uPA* transgene caused damage to the liver, mice were sacrificed at different ages and representative sections from the five liver lobes of experimental and control mice were processed for histologic examination. In the first two to three months after birth, transgenic liver sections did not show obvious damage as compared to liver samples from 2-month-old mice (Figure 1A). However, liver sections from mice that were 4 months or older showed necrotic nodules compared to younger mice (Figure 1B, 1C). Histologic studies on these mice showed increasing levels of inflammation and necrosis.

### Functionality of repopulated hepatocytes

**Figure 2:**
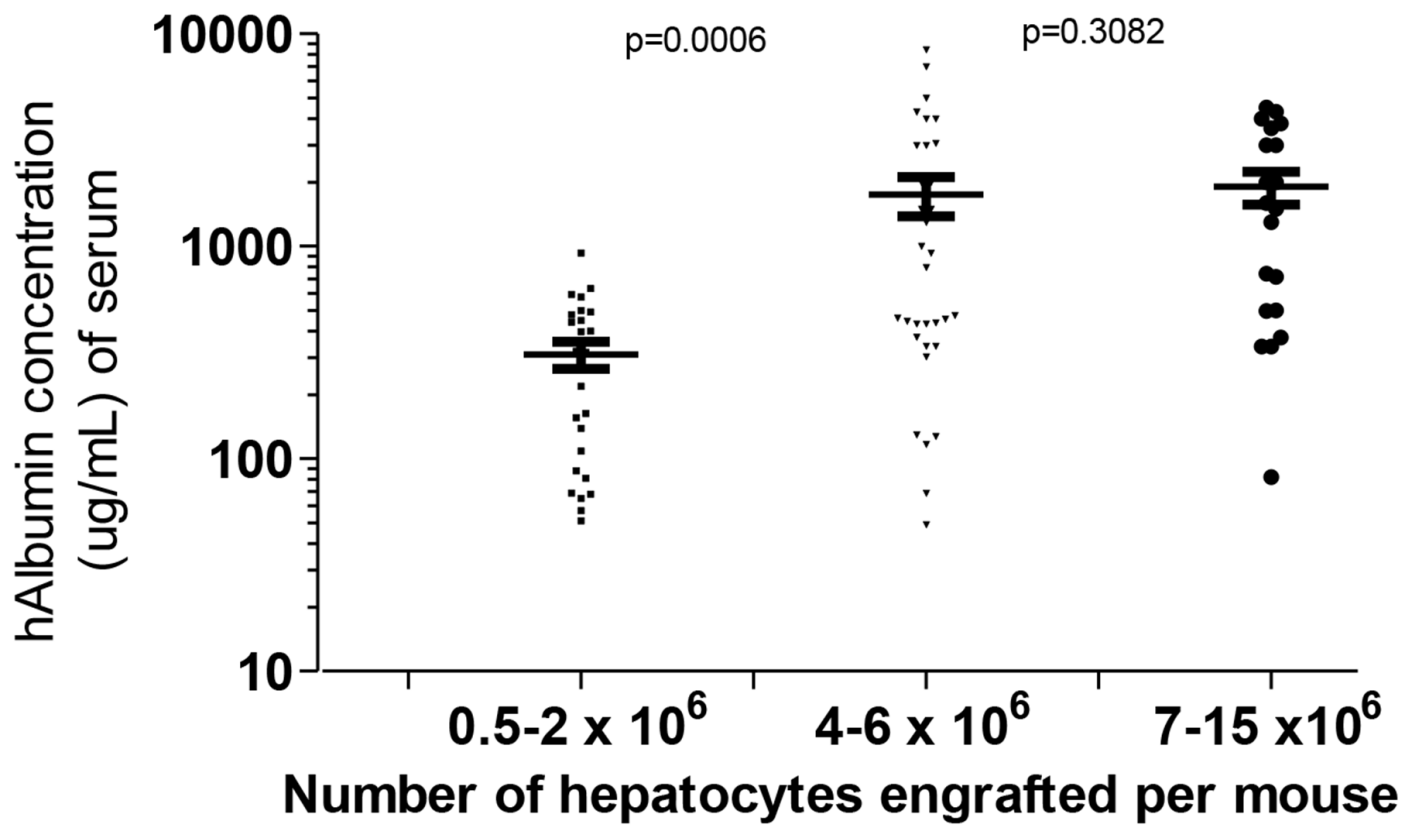
The number of human hepatocytes needed for optimum engraftment was determined by the level of human albumin secretion in the cells. Plots represent the mean human albumin concentration and interquartile ranges of human albumin levels (ug/mL) in the serum *of* *MUP-uPA-SCID/Bg* mice transplanted with different numbers of human hepatocytes (n=25, 310 ug/mL +45, n=31, 1.75 mg/mL +365, n=20, 1.8 mg/mL +339).

We determined the optimal number of human hepatocytes needed to achieve an adequate level of engraftment by increasing the number of cells used in the engraftment and assessed for the functionality of repopulated hepatocytes by measuring human albumin in the serum of the mice by ELISA 30 days after engraftment. While mice that were engrafted with a range of 0.5 x 10^6^ to 2.0 x 10^6^ hepatocytes expressed a mean of 320 ug/mL of human albumin, increasing the number of hepatocytes to 4-6 x 10^6^ cells per mouse significantly improved reconstitution and resulted in a mean of 1.9 mg/mL (***P= 0.0006) (Figure 2). Interestingly, further increase in cell numbers did not produce an increase in concentration of human albumin (1.8 mg/mL, P=0.3082) in the serum. 

### Detection and quantitation of human hepatocytes in transgenic mouse liver

**Figure 3:**
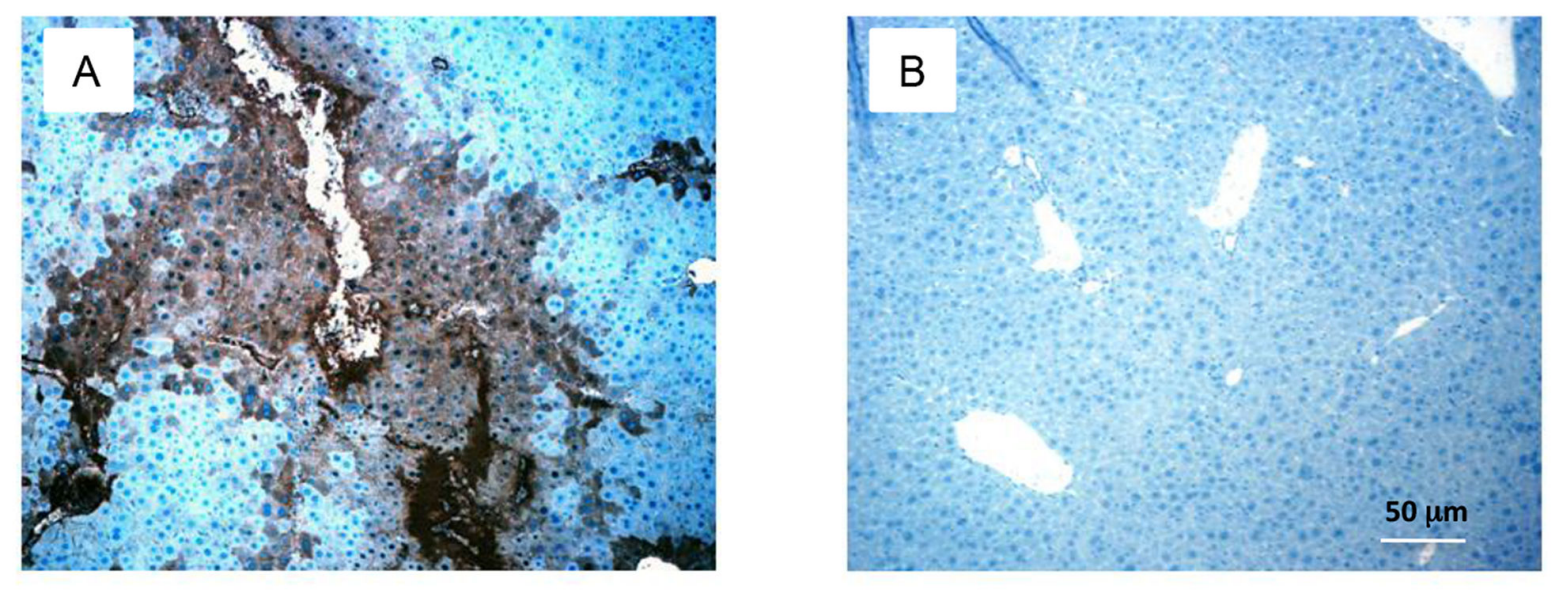
Engraftment of primary human hepatocytes into necrotic liver of *MUP-uPA/SCID/Bg* mice. **A**. Human hepatocytes are identified within the parenchyma of a chimeric mouse that had been engrafted with human hepatocytes at age 27 weeks and euthanized 10 weeks later. Staining was done with HRP-conjugated anti-human albumin (brown-black) and counter stained with hemotoxylin (blue). **B**. Section of liver from a control unengrafted transgenic mouse stained with similar HRP-conjugated anti- human albumin.

**Figure 4:**
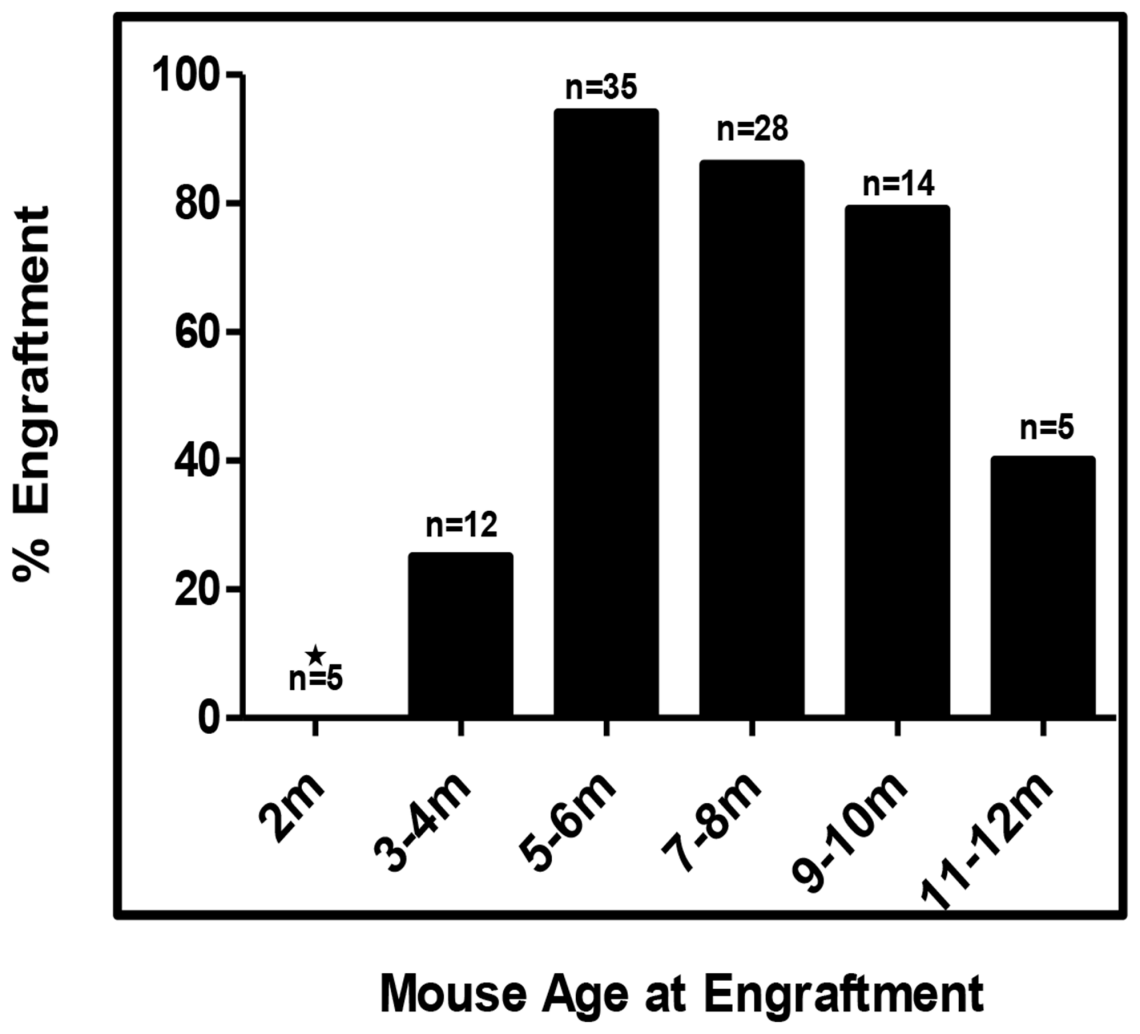
Percentage of mice successfully engrafted at various ages. One hundred mice at different ages at the time of engraftment with human hepatocytes were evaluated for successful engraftement by measuring human albumin concentration in the serum. Mice were considered successfully engrafted if we measured >100 ug/mL of human albumin.

To determine the extent of human hepatocytes engraftment in the liver of the mouse, we performed necropsies on mice sacrificed at various time points after engraftment. Liver from experimental mice showed extensive engraftment of functional human hepatocytes in areas of the diseased mouse liver. Production of human albumin was detected primarily in the diseased parenchyma and around the central vein. In addition, livers of these mice revealed large areas of mouse liver replaced by clusters of human hepatocytes that can be visualized with antibodies specific for human albumin. Figure 3 shows human albumin by immune-staining of a liver section from a mouse engrafted with human hepatocytes at age 27 weeks and euthanized 10 weeks later. Quantitation of hepatocyte engraftment was estimated at 20-40% in the human albumin stained liver samples from engrafted transgenic mice (n=50;***P < 0.0008) (Figure 4).

### Determination of optimal age of MUP-*uPA/SCID/Bg* mice for human hepatocyte engraftment

**Figure 5:**
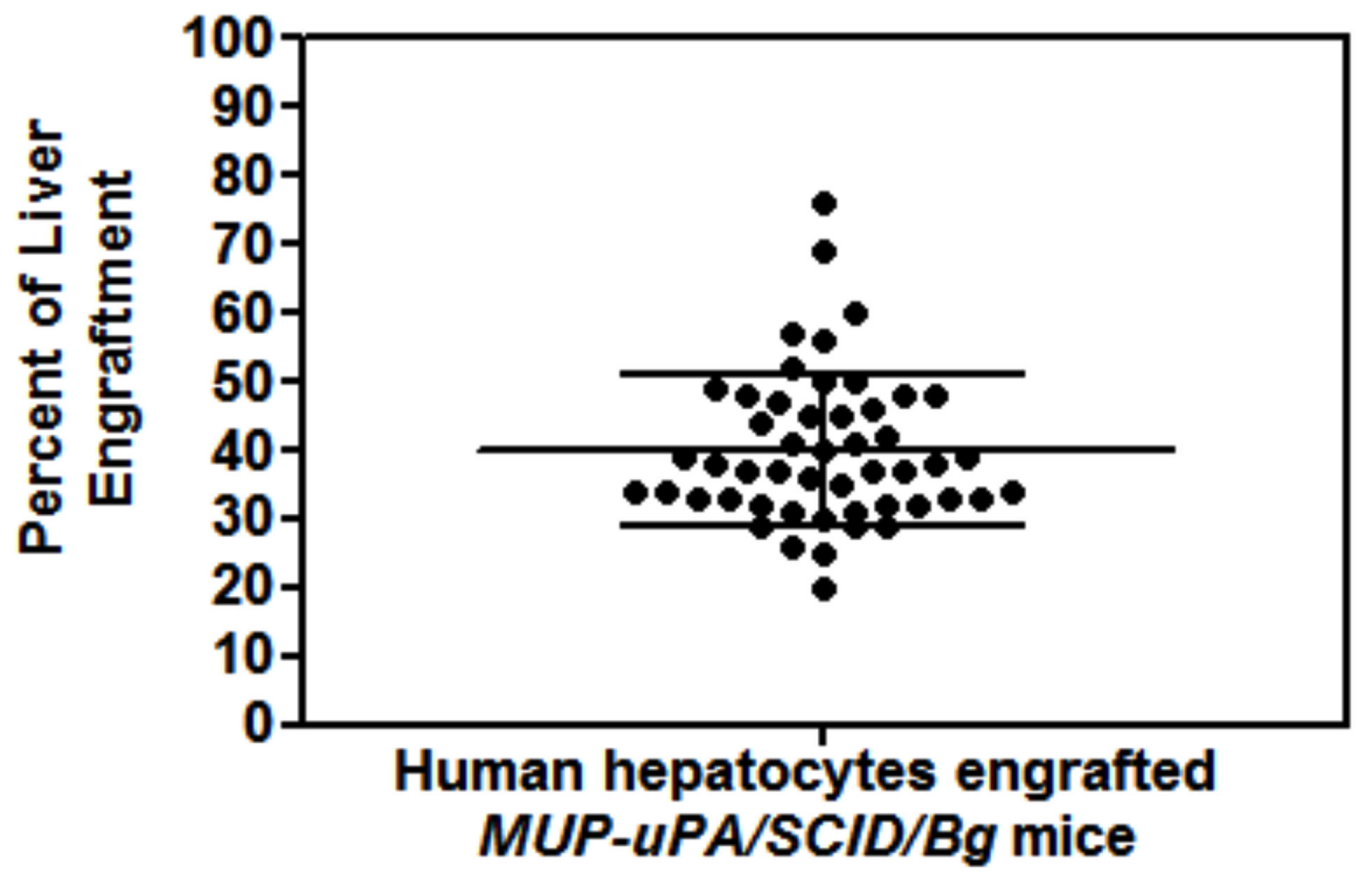
The level of human hepatocyte repopulation of transgenic mouse livers as determined by immuno-staining liver sections from human hepatocyte engrafted transgenic mice. Plot represents the mean and interquartile ranges of human albumin levels (ug/mL) in the serum *of* *MUP-uPA-SCID/Bg* mice transplanted with different numbers of human hepatocytes. (n=50; ***P < 0.0008).

We measured the serum human albumin concentrations about one month post-engraftment in one hundred mice that had been engrafted at ages varying from two to twelve months. We considered a mouse to have been successfully engrafted if the human albumin concentration exceeded 100 ug/mL as measured by ELISA. Figure 5 shows the percentage of successfully engrafted mice that were reconstituted with human hepatocytes at various age ranges up-to twelve months. Of the five mice engrafted before that age of two months of age, none had human albumin levels over 100 ug per mL of serum. 79% to 94% of mice were successfully engrafted between the ages of four and ten months, but the success rate dropped to 40% in mice that were eleven to twelve months of age. 

### HCV and HBV infection of chimeric mice

We inoculated 173 engrafted mice with HCV or HBV inocula and about 14% of these mice died spontaneously within a month of inoculation or they had to be euthanized due to poor health.

**Figure 6:**
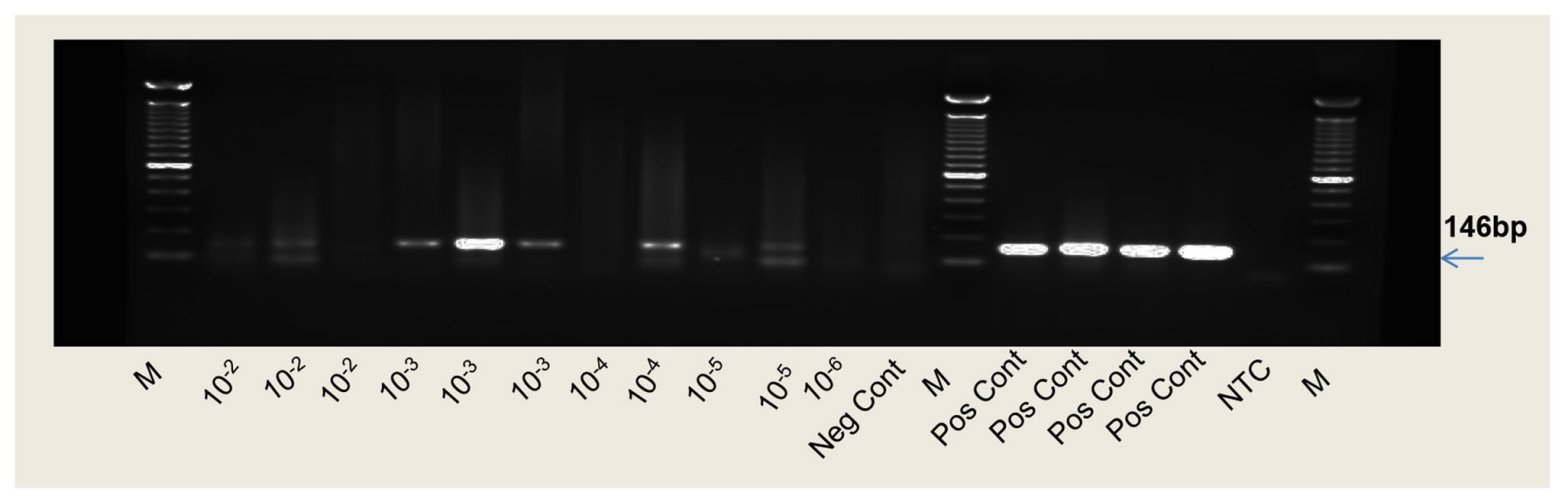
Sensitivity of *MUP-uPA/SCID/Bg* mice inoculated with HCV plasma derived from infected chimpanzee. Infection was determined by nested PCR on HCV RNA extracted from mouse sera. A 146bp nPCR product was detected by agarose gel electrophoresis. The lanes represent sera from mice inoculated serial 10-fold dilutions of an HCV infected chimpanzee serum that contained 10^4.5^ CID50/mL.

To test for the sensitivity to infection with HCV in the chimeric mice relative to the infectivity in chimpanzees , we inoculated intravenously (i.v.) a ten-fold dilution series of HCV (strain H) plasma containing a beginning titer of 10^4.5^ 50% chimpanzee infectious doses (CID_50_) per mL [27] into engrafted *MUP-uPA/SCID/Bg* mice (four mice per dilution) 30 days post engraftment. HCV replication was confirmed by nested PCR (Figure 6). While some mice died due to experimental manipulations, we tested 2 to 4 mice at each virus dilution in order to establish the lowest viral dose that induced a measurable infection. We then repeated the experiment with dilutions around the endpoint. We calculated a-50% endpoint infectivity [28] in these chimeric mice by testing the mouse sera by nPCR. We determined the mouse infectivity titer of this inoculum to be 1.3 x 10^4^ 50% mouse infectious doses (MID_50_) per mL compared to approximately 3 X 10^4^ CID50/mL in chimpanzee indicating that the mouse model has a similar sensitivity for HCV infection as the chimpanzee. 

**Figure 7:**
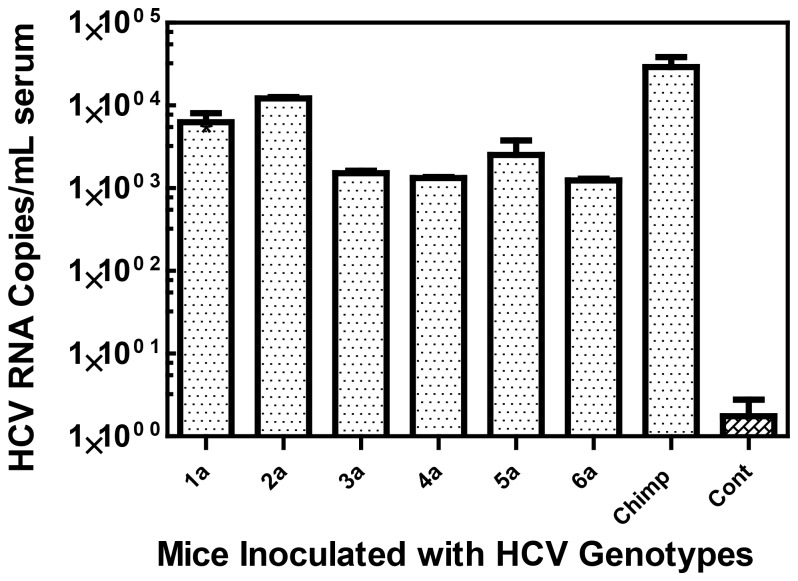
*MUP-uPA/SCID/Bg* mice infected with different HCV genotypes. The HCV RNA levels were measured by qRT-PCR on the serum of mice inoculated with various HCV genotypes (n=3-5).

**Figure 8:**
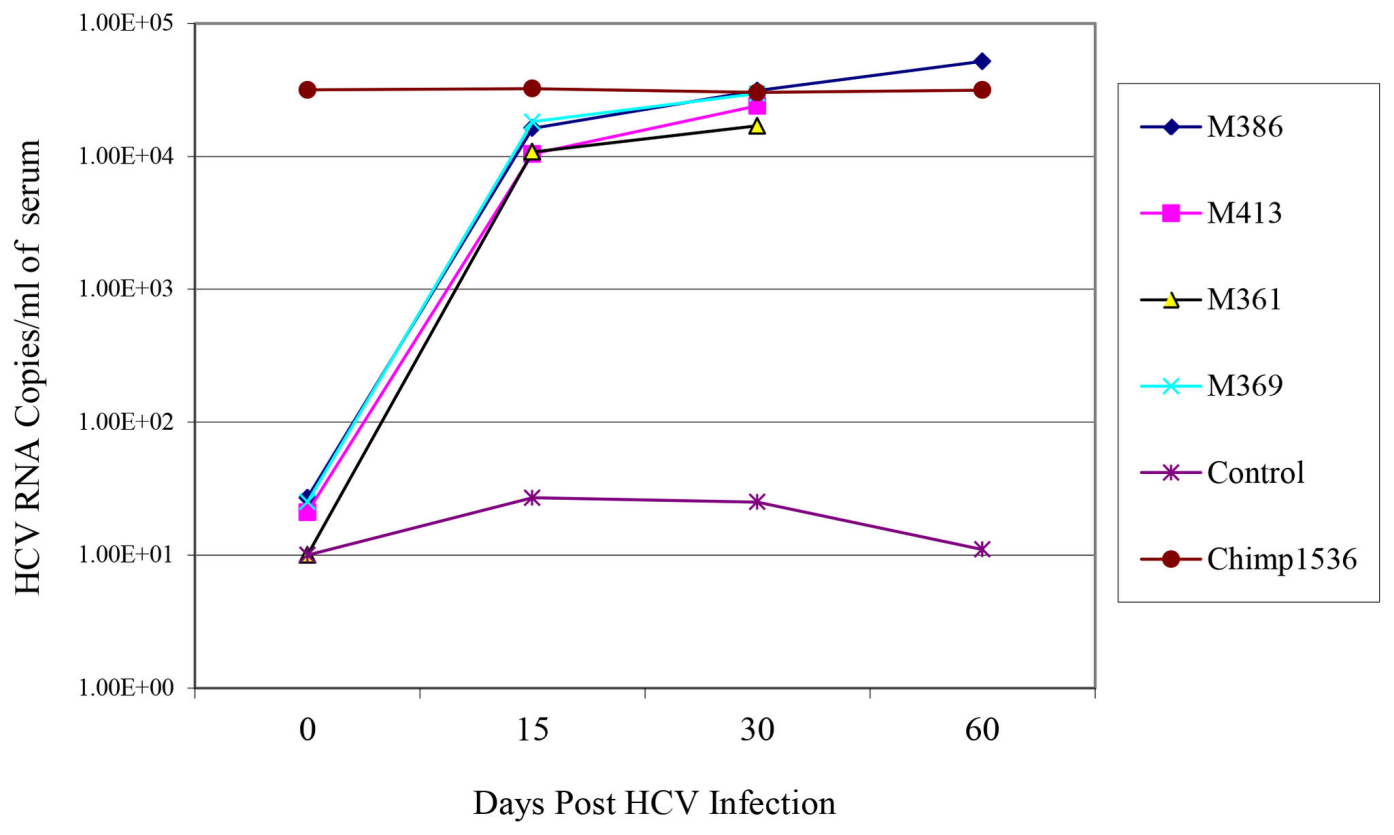
Long-term viral propagation of HCV genotype 1a in *MUP-uPA/SCID/Bg* chimeric mice. Several engrafted and HCV inoculated chimeric mice were monitored for 60 days post-inoculation for HCV replication and compared to mice that were not inoculated with HCV. Plasma from HCV-infected Chimpanzee was used as a positive control for HCV RNA copies in qRT-PCR assays.

In long-term follow-up of four genotype 1a-infected mice, we observed HCV RNA titers between 10^3^ to 10^4^ RNA copies per mL of serum. The RNA remained detectable for up to 2 months after inoculation (Figure 7).In order to determine whether these mice were susceptible to other HCV genotypes (GT), mice were inoculated with 100 CID_50_ of HCV isolates for GT-1a, 2a, 3a, 4a, 5a, and 6a that had been titered in chimpanzees [22]. The mouse infectivity of each of these inocula was determined by nested -PCR on the serum of each inoculated mice (Table 2). The resultant HCV RNA titers in the infected mice were determined by testing sera from each of the infected mice by qRT-PCR and averaging the results of all the infected mice inoculated with the same genotype (Figure 8). HCV RNA titers of 10^3^ to 10^5^ copies per ml of serum were measured in mice infected with different HCV genotypes (Figure 8). 

**Table 2:** *MUP-uPA/SCID/Bg* mice infected with different HCV genotypes.

HCV Strain	Genotype	Chimpanzee	Infected mice/
		Infectious Dose*	Total inoculated mice**
HC-TN	1a	100	4/4
HC-J6	2a	100	4/4
S52	3a	100	5/5
ED43	4a	100	6/6
SA13	5a	100	3/6
HK-6a	6a	100	5/5

* 100 CID50 in 100 uL of HCV plasma was used to infect human hepatocyte-engrafted mice.

** Serum from infected mice was tested for HCV RNA by nPCR.

Finally, we tested the susceptibility of the chimeric mice to infection by the cell culture adapted genotype 2a HCV, J6/JHF1. We inoculated 200 uL of J6/JFH1 infected Huh-7.5 cell culture supernatant into the spleens of two chimeric mice. Both mice were infected and HCV RNA titers of 1 x 10^5^ RNA copies per mL of serum, was achieved (data not shown).

Using similar methodology, we also tested transgenic mice for susceptibility to infection with HBV. We used a serum from a chronic HBV infected chimpanzee with an infectivity titer of 10^7.5^ CID_50_ per mL [28]. We tested the mouse infectivity of this serum by making serial 10-fold dilutions from10^-2^ to 10^-8^ and inoculated 4 to 6 mice with 100 uL for each dilution. The HBV MID_50_ was calculated by the Reed-Muench method to be 1.4 x 10^5^ per mL. 

**Figure 9:**
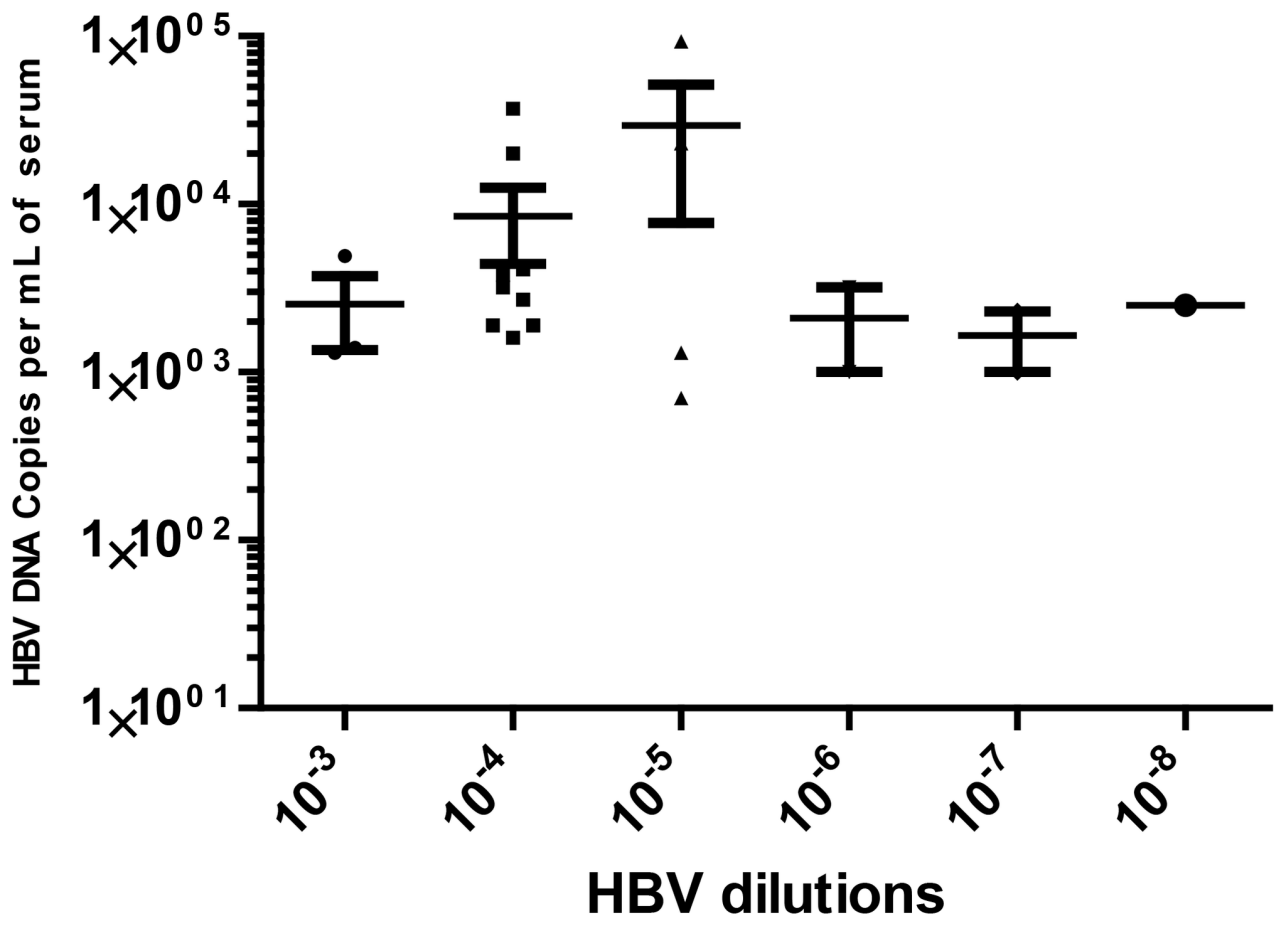
*MUP-uPA/SCID/Bg* mice sensitivity to HBV. Titration of hepatitis B virus in mice infected with various virus dilutions of HBV-infected chimpanzee plasma and assayed by q-PCR (n= 3-5).

Similar to the HCV experiment, we quantified the HBV DNA in the sera of infected mice by q-PCR. The mean serum level of HBV DNA from each titration group ranged between 10^3^ and 10^5^ but did not correlate with the inoculum dose. The highest titers were observed in mice inoculated with the 10^-4^ and 10^-5^ dilutions while all the others had similar, but lower levels of HBV DNA copies (Figure 9). 

In order to test for liver pathology associated with viral infection, we measured the serum levels of alanine aminotransferase (ALT) in mice infected with either HBV (n=3) or HCV (n=23) as well as two control mice. The measured ALT levels were generally very low, but highly variable and did not show any correlation with the level of serum human albumin or viral infection (data no shown).

### Immunohistochemical staining of liver samples from HCV and HBV inoculated transgenic mice

**Figure 10:**
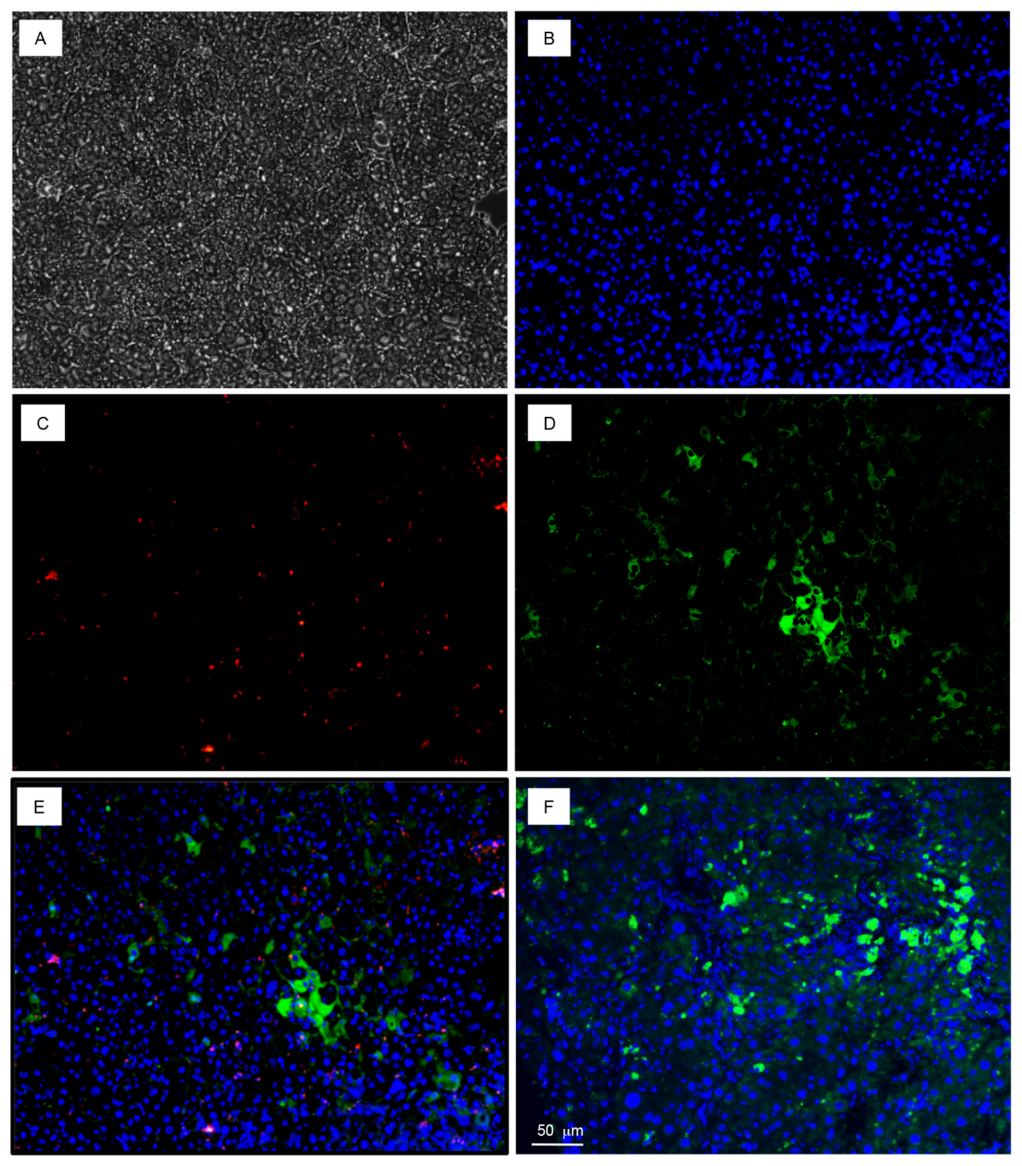
Detection of HCV infection in transgenic MUP-uPA/SCID/Bg mice engrafted with human hepatocytes. **A**. .Bright field image of transgenic mouse liver section. **B**. DAPI staining of nuclei. **C**. Stained with anti-hAlb (green). **D**. Stained with anti-HCV (NS5) antibody (red). **E**. Merged sections that were stained with both anti-hAlb and anti-HCV (NS5) primary antibodies (yellow). **F**. Section from engrafted but not virus infected mouse liver stained with anti-hAlb and anti-HCV (NS5) primary antibodies.

**Figure 11:**
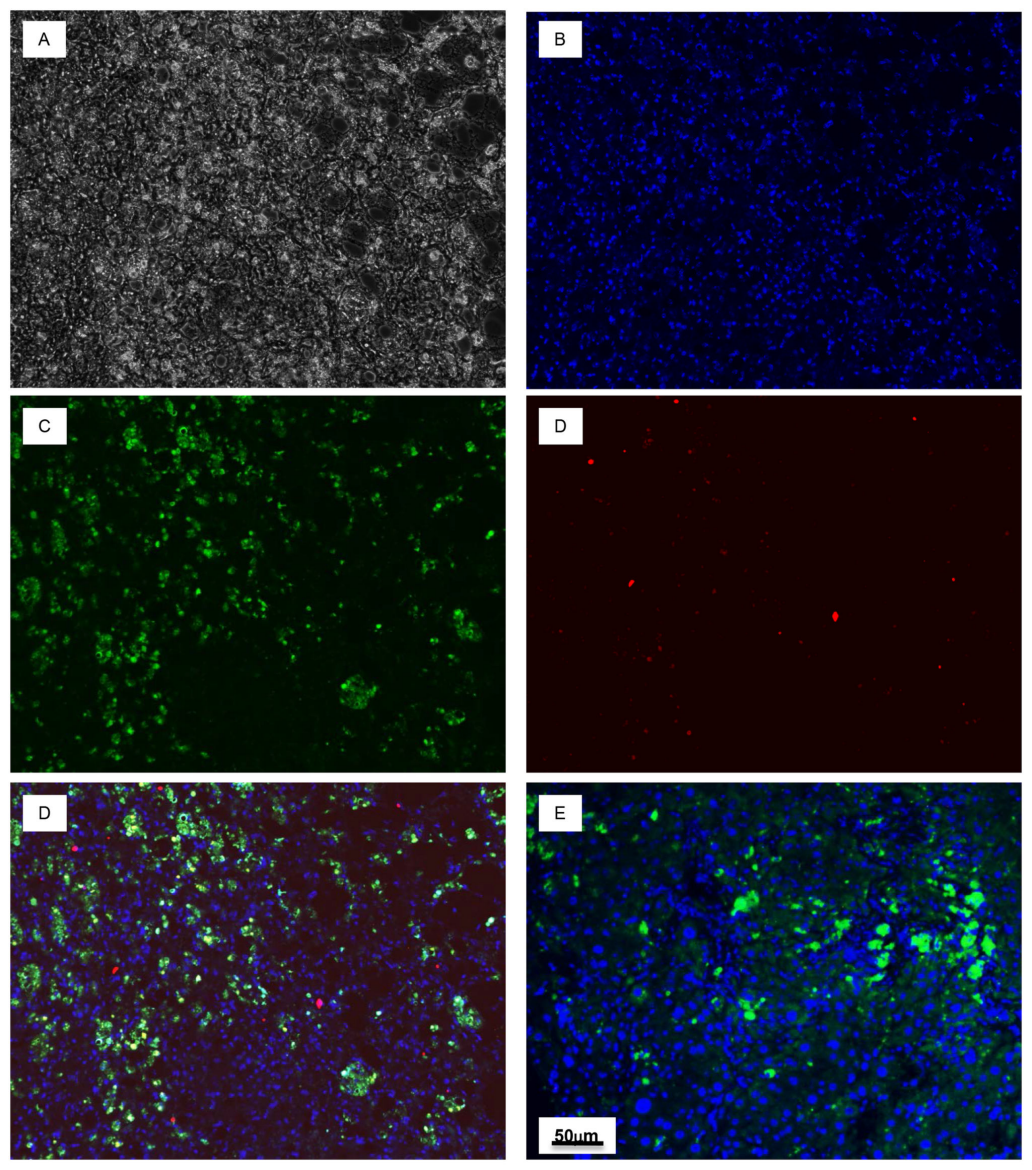
Detection of HBV infection in transgenic *MUP-uPA/SCID/Bg* mice engrafted with human hepatocytes. **A**. Bright field image of transgenic mouse liver section. **B**. DAPI staining of nuclei. **C**. Stained with anti-hAlb (green). **D**. Stained with primary anti-HBV (core Ag) antibody (red). **E**. Merged sections that were stained with both anti-hAlb (green) and anti-HBV (core Ag) primary antibodies (yellow). **F**. Section from engrafted but not virus inoculated mouse liver stained with anti-hAlb and anti-HBV (core Ag) primary antibodies.

Chimeric mouse liver samples were immunostained with fluorescent dyes for human albumin, HCV (NS5) or hepatitis B virus core antigen (HBcAg) and mounted in Ultra Cruz mounting medium (Santa Cruz Biotechnology) with 4’,6-diamidino-2-phenylindole (DAPI) for nuclear stain. Hepatocyte clusters showed cytoplasmic localization for human albumin and HCV (NS5) or human albumin and HBcAg (Figures 10 and 11). In contrast, samples from human hepatocyte engrafted mice, but not infected with HCV or HBV stain for only the human albumin marker. Samples from engrafted and infected mice stained negative when incubated with irrelevant polyclonal antibodies against H1N1influenza virus A or HIV-1 and the appropriately- labeled secondary antibody (data not shown).

## Discussion

Rapid advancements to our understanding of the virology, immunology and pathogenesis of hepatitis C have occurred over the past two decades with the use of the available scientific tools. Even before the discovery of the virus cause of hepatitis C, much had been learned about the disease from the study of patients but even more so through the use of the chimpanzee model for HCV infection [6]. While the use of chimpanzees for this research has been invaluable, this model also had many limitations that include expense, availability and ethical considerations. A recent review by the National Academy of Science has placed the chimpanzee in a special category of research animals and at the present, chimpanzee research is severely restricted [29]. Therefore, the need for a small animal model for HCV research has become even more important.

In 2001, Mercer and colleagues reported an immunodeficient transgenic mouse into which human liver had been successfully engrafted [12]. These mice were shown to support the replication of human hepatitis B and C. The basis of this system is the use of a transgenic mouse expressing the gene urokinase plasminogen activator (uPA) under control of the strong liver specific albumin promoter in a highly immunodeficient mouse background. The uPA expression results in the destruction of the mouse liver and when human liver is then engrafted, it replaces the diseased mouse liver under the signals for regeneration being elaborated by the dying mouse hepatocytes. Human hepatitis viruses are then able to replicate in the human hepatocytes. As these mice are immunodeficient, they are not useful for many kinds of studies but this type of model is still valuable for investigations in which virus replication is the primary object of the experiment.

While this system has been extremely useful for a variety of studies, *Alb-uPA* mice are very difficult to work with and are not practical for most laboratories. Unless breeding mice are engrafted with normal liver, the mouse colony must be maintained as hemizygotes for the uPA transgene as the dizygotic mice are generally too impaired to breed well [15]. There is a high neonatal fatality rate among the dizygotes, but because the hemizygotic mice do not accept the graft efficiently dizygotes must be bred from hemizygotic breeders for each experiment. As the transgene is expressed from birth the mice must be engrafted with the first few weeks of life resulting in a high experimental death rate.

Several alternative mouse models such as the *Fah* ^*–*^ */* ^*–*^ */Rag2* ^*–*^ */* ^*–*^ */Il2rg* ^*-*^ */* ^*-*^ (FRG) mice [17,18],, show high infectivity based on robust repopulation of the mouse liver [19]. However, the mice must be maintained on the drug 2-(2-nitro-4-trifluromethylbenzoyl)-1, 3-cyclohexanedione (NTBC) until they are ready to engraft and withdrawal of the drug may result in very severe liver disease.

In this paper, we describe a relatively simple mouse model that would be useful for any laboratory capable of maintaining immunodeficient mice. The *MUP-uPA/SCID/Bg* model solves some of the problems associated with the *Alb-uPA* mice [7]. The mice described in this study utilize the same *uPA* transgene behind the major urinary protein promoter (*MUP*) and crossed onto *SCID/beige* background (*MUP-uPA/SCID/Bg*) [21]. We looked at multiple liver sections from the *MUP-uPA SCID/Bg* mice between 2 and 6 months of age for evidence of hepatic destruction. We found visible necrosis only on liver samples from mice over 4 month or older. From these observations, we concluded that we could engraft these mice over a prolonged window of time, which would reduce technical problems relating to the size or age of mice. These *MUP-uPA* transgenic mice that are injected with human hepatocytes generally repopulate over 40% of the mouse liver as evidenced by immuno-histochemical staining of the engrafted livers with antibodies against human albumin. Routinely, HCV RNA replication as measured by real-time quantitative PCR on the order of 10^3^ to 10^5^ copies of HCV RNA per mL of serum in 80% of engrafted and inoculated *MUP-uPA* transgenic mice. *MUP-uPA* chimeric mice were inoculated with 100 CID_50_ of different genotypes of the HCV, 1a, 2a, 3a, 4a, 5a and 6a obtained from patients and passed in chimpanzees [22]. Although the viral replication in these chimeric mice varied, each of the representatives of the major HCV genotypes was infectious in the *MUP-uPA* chimeric mice. The system also supported hepatitis B virus (ayw subtype) replication though it was not extensively studied.

These *MUP-uPA/SCID/Bg* transgenic mice, even without human liver engraftment, are healthy enough to survive at least to one year after birth and provide a window from about 4 months and continuing to at least one year of age for engraftment with human hepatocytes and infection with hepatitis viruses. As the mice are adult at the time of engraftment, the surgical procedure is far easier and experimental deaths are rare.

The *MUP-uPA/SCID/Bg* mice remain dizygous for the *uPA* transgene for several generations and there are no problems in maintaining a colony of transgenic mice that will be available for experimental procedures at any time. In our experiments to date, we have had only rare early neonatal deaths, and while we had an overall 9% surgical mortality, most of those occurred in the early experiments while we were improving the surgical techniques. The procedure for liver engraftment is optimized such that it requires very simple technical skills and takes about 5 minutes per mouse most of which is used for the induction of anesthesia. There is about 20-40% engraftment of human hepatocytes by immunohistochemistry staining for human albumin in these mice. We have observed a correlation between the number of human hepatocytes inoculated and the engraftment level as measured by the concentration of human albumin in the serum. 

We have shown the *MUP-uPA* transgenic mice can be reproducibly engrafted and infected with HBV and the six genotypes of HCV. All the mice inoculated with the specific genotype chimpanzee plasmas were infected except for those inoculated with genotype 5a. Of the six mice inoculated with HCV GT 5a, only 3 were infected as determined by the nested RT-PCR. It is possible that this 5a strain was less infectious in our mice. It is also possible that the 5a inoculum itself was relatively less infectious compared to the other genotypes as the chimpanzee titer was determined by reverse titration using ten-fold dilution in just one chimpanzee for each genotype and the true infectivity could fit within a rather broad range [7]. It is also possible that our PCR was less sensitive for the genotype 5a sequence but this was not evaluated. We did show that the average HCV RNA titer (determined by qRT-PCR) in the three GT 5a infected mice was about the same as in the mice infected with the other five genotypes.

In addition to the clinical isolates of HCV, we have also infected these chimeric mice with tissue-culture derived HCV (HCV J6/JFH1) (data not shown). Therefore, these chimeric mice may also be useful for the study of mutant viruses made using the cell culture adapted viruses. 

In this study, we observed a direct correlation of the extent of human hepatocyte reconstitution with increasing numbers of viable human hepatocytes that are inoculated in the *MUP-uPA/SCID/Bg* mice. In order to achieve a good level of engraftment, we found that between 4 X10^6^ and 6 X10^6^ viable human hepatocytes per mouse was about optimal. 

Although there was some variability in the viability of cells due to the donor source, we have established a protocol for the optimal time points for engraftment and viral infection post engraftment which will allow for experiments to be performed routinely and reproducibly. As in all the mouse model systems using immunodeficient mice, the use of the *MUP-uPA/SCID/Bg* system has limitations for studies involving the immune response to HCV infections. However, we are encouraged that these mice are a useful improvement that overcome some of the problems associated with the presently available *Alb/uPA* mice. 

Future work in the mouse models could include the development of immune-competent mice [30-32] that would more completely model HCV infection and disease. Such mice would be very useful for the study in the immune response to HCV infections, immunopathogenesis and possibly vaccine development. Nevertheless, the immune-deficient mice are useful in many experiments in which a read-out of viral infection is the endpoint. We have shown that our mouse model has a similar sensitivity to HCV infection as the chimpanzee including wild type HCV strains and therefore could replace the chimpanzee for these experiments. Each of the published chimeric mouse models has advantages and disadvantages. The system that we have described in this paper is both robust and simple enough to implement that it is practical for use in many laboratories even though it may not yield titers as high as have been reported with other mouse models. 
